# Fluoxetine and Vortioxetine Reverse Depressive-Like Phenotype and Memory Deficits Induced by Aβ_1-42_ Oligomers in Mice: A Key Role of Transforming Growth Factor-β1

**DOI:** 10.3389/fphar.2019.00693

**Published:** 2019-06-21

**Authors:** Sebastiano Alfio Torrisi, Federica Geraci, Maria Rosaria Tropea, Margherita Grasso, Giuseppe Caruso, Annamaria Fidilio, Nicolò Musso, Giulia Sanfilippo, Fabio Tascedda, Agostino Palmeri, Salvatore Salomone, Filippo Drago, Daniela Puzzo, Gian Marco Leggio, Filippo Caraci

**Affiliations:** ^1^Department of Biomedical and Biotechnological Sciences, University of Catania, Catania, Italy; ^2^Department of Drug Sciences, University of Catania, Catania, Italy; ^3^Oasi Research Institute—IRCCS, Troina, Italy; ^4^Bio-nanotech Research and Innovation Tower (BRIT), University of Catania, Catania, Italy; ^5^Department of Life Sciences and Center for Neuroscience and Neurotechnology, University of Modena and Reggio Emilia, Modena, Italy

**Keywords:** Alzheimer’s disease, amyloid-β, vortioxetine, antidepressants, fluoxetine, memory, TGF-β1, depression

## Abstract

Depression is a risk factor for the development of Alzheimer’s disease (AD), and the presence of depressive symptoms significantly increases the conversion of mild cognitive impairment (MCI) into AD. A long-term treatment with antidepressants reduces the risk to develop AD, and different second-generation antidepressants such as selective serotonin reuptake inhibitors (SSRIs) are currently being studied for their neuroprotective properties in AD. In the present work, the SSRI fluoxetine and the new multimodal antidepressant vortioxetine were tested for their ability to prevent memory deficits and depressive-like phenotype induced by intracerebroventricular injection of amyloid-β (1-42) (Aβ_1-42_) oligomers in 2-month-old C57BL/6 mice. Starting from 7 days before Aβ injection, fluoxetine (10 mg/kg) and vortioxetine (5 and 10 mg/kg) were intraperitoneally injected daily for 24 days. Chronic treatment with fluoxetine and vortioxetine (both at the dose of 10 mg/kg) was able to rescue the loss of memory assessed 14 days after Aβ injection by the passive avoidance task and the object recognition test. Both antidepressants reversed the increase in immobility time detected 19 days after Aβ injection by forced swim test. Vortioxetine exerted significant antidepressant effects also at the dose of 5 mg/kg. A significant deficit of transforming growth factor-β1 (TGF-β1), paralleling memory deficits and depressive-like phenotype, was found in the hippocampus of Aβ-injected mice in combination with a significant reduction of the synaptic proteins synaptophysin and PSD-95. Fluoxetine and vortioxetine completely rescued hippocampal TGF-β1 levels in Aβ-injected mice as well as synaptophysin and PSD-95 levels. This is the first evidence that a chronic treatment with fluoxetine or vortioxetine can prevent both cognitive deficits and depressive-like phenotype in a non-transgenic animal model of AD with a key contribution of TGF-β1.

## Introduction

Alzheimer’s disease (AD) is a neurodegenerative disorder characterized by memory loss, cognitive decline, and neuropsychiatric symptoms, such as depression and psychotic signs, which strongly interfere with normal daily activities (Lanctôt et al., 2017). Different neurobiological and clinical links have been found between depression and AD (Caraci et al., 2018). Depression is a risk factor for the development of AD, and the presence of depressive symptoms significantly increases the conversion of mild cognitive impairment (MCI) into AD (Modrego and Ferrández, 2004). Common pathophysiological events have been identified in depression and AD, including activation of the hypothalamic–pituitary–adrenal (HPA) axis with increased glucocorticoid levels, neuroinflammation with an aberrant tumor necrosis factor-α (TNF-α) signaling, and an impairment of transforming growth factor-β1 (TGF-β1) signaling (Caraci et al., 2018).

Intracerebroventricular (i.c.v.) injection of oligomers of amyloid-β (1-42) (Aβ_1-42_), the most toxic form of amyloid aggregates in AD brain, can induce both memory deficits and depressive-like phenotype in rats (Colaianna et al., 2010; Schiavone et al., 2017) and mice (Ledo et al., 2013; Ledo et al., 2016), while an acute treatment with the selective reuptake inhibitor (SSRI) fluoxetine can revert this phenotype (Ledo et al., 2013; Ledo et al., 2016; Schiavone et al., 2017). Evidence also exists that fluoxetine prevents amyloid pathology and reverses memory impairment in different AD animal models (Wang et al., 2014; Jin et al., 2016). Interestingly, a continued long-term treatment with antidepressants is known to reduce the risk to develop AD (Kessing et al., 2009; Kessing, 2012). It has been hypothesized that a chronic treatment with second-generation antidepressants can exert relevant neuroprotective effects in depressed MCI patients with a high risk to develop AD, but the molecular mechanisms underlying the neuroprotective effects of antidepressants are not yet completely understood (Caraci et al., 2018).

Deficit of TGF-β1 signaling is a common pathophysiological event in both depression and AD (Caraci et al., 2018). Among SSRIs, fluoxetine increases circulating TGF-β1 levels in depressed patients (Lee and Kim, 2006; Sutcigil et al., 2007) and prevents Aβ-induced toxicity in neuronal cultures by increasing the release of TGF-β1 (Caraci et al., 2016). However, it is presently unknown whether a chronic treatment with fluoxetine or other second-generation antidepressant drugs can prevent memory deficits and depressive-like phenotype in animal models of AD.

Vortioxetine is a third-generation antidepressant with a novel, multimodal, mechanism of action, directly acting on several serotonin (5-hydroxytryptamine, 5-HT) receptors (as an agonist on 5-HT_1A_ receptor, a partial agonist on 5-HT_1B_, and an antagonist on 5-HT_1D_, 5-HT_3_, and 5-HT_7_) besides inhibiting the serotonin transporter (SERT; MØrk et al., 2012). Several preclinical studies have clearly demonstrated robust pro-cognitive effects of vortioxetine in different animal models of depression (Pehrson et al., 2015). In particular, vortioxetine displays a superior efficacy on visuospatial memory and depressive-like behavior, than does fluoxetine, in aged mice (Li et al., 2015; Li et al., 2017). Recent clinical studies also suggest an improved efficacy of vortioxetine on specific clinical domains, where SSRIs are less effective, such as cognitive deficits associated with major depressive disorder (MDD; Thase et al., 2016), in particular in elderly patients (McIntyre et al., 2016).

No studies have been conducted so far to examine the preclinical efficacy of vortioxetine compared with fluoxetine in treating depressive-like behavior and memory impairment induced by the i.c.v. injection of Aβ_1-42_ oligomers.

The aim of the present study is to assess whether a chronic treatment with fluoxetine or vortioxetine can prevent memory deficits and depressive-like phenotype in a non-Tg model of AD obtained by i.c.v. injection of Aβ_1-42 _oligomers.

We show that a chronic (24 days) treatment with fluoxetine or vortioxetine in young (2-month-old) C57BL/6 mice can revert both Aβ-induced depressive-like behavior and memory impairment with a key contribute played by TGF-β1.

## Materials and Methods

### Animals

Eight-week-old male C57BL/6 mice, from Envigo RMS s.r.l. laboratories (San Pietro al Natisone, Italy), were individually housed, with free access to chow and water, in an air-conditioned room, with a 12-h light–dark cycle and with constant temperature (23 ± 1°C) and humidity (57 ± 3%) conditions. Animals were left undisturbed for 1 week before beginning any behavioral procedure. All animal experiments were carried out in accordance with Italian (D.M. 116192) and EEC (O.J. of E.C.L 358/1 12/18/1986) regulations on protection of animals. Every effort has been made to minimize animal suffering and to reduce the number of animals used.

### Preparation of Human Aβ_1-42_ Oligomers and i.c.v. Injection in Mice

Synthetic human Aβ_1-42_ oligomers were prepared according to the original protocol of Klein’s group (Gong et al., 2003). Briefly, the Aβ_1-42_ lyophilized peptide, purchased from Bachem Distribution Services GmbH (Weil am Rhein, Germany), was dissolved in trifluoroacetic acid (TFA) (1 mg/ml) and sonicated in a water bath sonicator for 10 min. Then, TFA was evaporated under a gentle stream of argon, and 1 ml of 1,1,1,3,3,3-hexafluoro-2-propanol (HFIP) was added to the peptide. After 1-h incubation at 37°C, the peptide solution was dried under a stream of argon and then solubilized again by adding 2 ml of HFIP. Finally, HFIP was removed by argon streaming followed by further drying in a lyophilizer for 1 h, and then Aβ_1-42_ was suspended in 5 mM of anhydrous dimethyl sulfoxide (DMSO), before dilution to 100 μM in ice-cold cell culture medium Dulbecco's Modified Eagle Medium/Nutrient Mixture F-12 (DMEM/F12). Samples of Aβ_1-42_ at the concentration of 100 μM were incubated for 72 h at 4°C and then stored at −20°C until use.

To obtain a non-transgenic (non-Tg) AD model, animals were anesthetized for 7 min with 2.5% isoflurane using a vaporizer system and gently restrained only during the injection procedure. Aβ_1-42_ oligomers were administered i.c.v. into the brain. Synthetic human Aβ_1-42_ oligomers were diluted from the stock in DMEM solution (100 μM) in sterile 0.1 M phosphate buffered saline (PBS) (pH 7.4) at a final concentration of 10 µM and then injected i.c.v. Sterile 0.1 M PBS was injected i.c.v. into control animals (vehicle). Intracerebroventricular injection was used because of its simplicity with respect to stereotaxis in mice and to ensure diffusion of Aβ_1-42_ in the whole brain (Maurice et al., 1996; Leggio et al., 2016). Two microliters was injected using a microsyringe with a 28-gauge 3.0-mm-long stainless steel needle (Hamilton); 2 µL of the 10 µM Aβ solution corresponds to 20 pmol of Aβ monomer equivalent, e.g., 0.09 µg Aβ per mouse brain (weighing around 500 mg). Assuming that soluble Aβ oligomers are freely diffusing in cerebrospinal fluid and then in the brain, their final concentration would be approximately 0.18 µg/g of tissue.

### Drugs and Treatment

Vortioxetine hydrobromide [purity > 98.0% (HPLC)] was provided by H. Lundbeck A/S (Denmark) according to the MTA N.417394 signed by University of Catania (Department of Drug Sciences) and H. Lundbeck A/S and Lundbeck Italia S.p.A. Fluoxetine hydrochloride [product number: F132; purity > 98.0% (TLC)] was purchased from Sigma-Aldrich (St Louis, MO). Both compounds were dissolved in DMSO and further diluted with a final concentration of 1% of DMSO. Fluoxetine was administered intraperitoneally (i.p.) at the dose of 10 mg/kg (100 µL/10 g body weight), while vortioxetine was administered i.p. at two different doses (5 and 10 mg/kg; 100 µL/10 g body weight). Control animals received the vehicle i.p. (100 µL/10 g, DMSO 1%). The fluoxetine dose and the two vortioxetine doses were selected on the basis of previous studies where these antidepressant drugs were administered in animal models of depression (Pehrson et al., 2015).

### Experimental Design

In order to assess the effects of fluoxetine and vortioxetine on depressive-like behavior and memory impairment induced by Aβ oligomers, three different cohorts of animals were used, according to the following experimental design.

*Experiment 1 (first cohort):* No i.c.v. injection of Aβ_1-42_ oligomers was performed in this cohort. Mice were randomly divided into four experimental groups (*n* = 7–10 mice per treatment group): vehicle, fluoxetine (FLX) 10 mg/kg, vortioxetine (VTX) 5 mg/kg, and VTX 10 mg/kg. All drugs were administered i.p. for 21 days. To assess the antidepressant activity of fluoxetine and vortioxetine, mice were tested in the forced swim test (FST) on day 22.

*Experiment 2 (second cohort):* Aβ_1-42_ oligomers or PBS i.c.v. injection was performed in this cohort of mice 7 days after the beginning of antidepressant treatment (day 7). The treatment with antidepressants lasted until day 26, when all behavioral tests were completed. Mice were randomly allocated to five experimental groups (*n* = 7–8 animals/group): PBS i.c.v. + vehicle i.p., Aβ i.c.v. + vehicle i.p., Aβ i.c.v. + FLX 10 mg/kg i.p., Aβ i.c.v. + VTX 5 mg/kg i.p., and Aβ i.c.v. + VTX 10 mg/kg i.p. Memory deficits were evaluated after 24 days of chronic treatment with FLX or VTX in the passive avoidance test (PAT), 15–17 days after Aβ injection, whereas depressive-like behavior was evaluated with FST after 26 days of treatment with antidepressant drugs.

*Experiment 3 (third cohort):* Animals received 3 weeks of treatment with antidepressants and Aβ_1-42_ oligomers or PBS. Intracerebroventricular injection was performed 7 days after the beginning of antidepressant treatment (day 7). Experimental groups were not only those described in Experiment 2 but also those included the following four experimental groups: vehicle, FLX 10 mg/kg, VTX 5 mg/kg, and VTX 10 mg/kg. This third cohort of animals was tested in the object recognition test (ORT), after 21 days of chronic treatment with FLX or VTX.

### Forced Swim Test

The FST protocol employed here was adapted from Porsolt et al., (1978). Mice were placed for 6 min in a 4-L Pyrex glass beaker containing 3 L of water at 24 ± 1°C. Water was changed between animals. After a habituation period of 2 min, mobility and immobility were recorded during the last 4 min of the 6-min testing period. A trained researcher blinded to group assignment recorded immobility time using a stopwatch. An increase in immobility time indicates depressive-like behavior. A mouse was judged immobile when it floated in an upright position and displayed only small movements to keep its head above water.

### Passive Avoidance Test

PAT was performed as previously described (Leggio et al., 2016). The apparatus for the step-through PAT was an automated shuttle box divided into an illuminated compartment and a dark compartment of the same size by a wall with a guillotine door. In the experimental session, each mouse was trained to adapt to the step-through passive avoidance apparatus. In the adaptation trial, the animal was placed into the illuminated compartment. After 10 s, the door between these two boxes was opened, and the mouse was allowed to freely move into the dark compartment. The learning trial was similar to the adaptation trial except that the door was closed automatically as soon as the mouse stepped into the dark compartment and an inescapable foot shock (0.2 mA, 2 s) was delivered through the grid floor. Following the shock, the mouse was removed and returned to its home cage. The retention of the step-through passive avoidance response was measured the day after the learning trial, and the latency to re-enter into the dark compartment was recorded. In the retention test, no foot shock was delivered. Adaptation trial, learning trial, and retention test were performed 15, 16, and 17 days, respectively, after PBS or Aβ i.c.v. injections (see above for details regarding the experimental design).

### Object Recognition Test

ORT was performed as previously described (Gulisano et al., 2018). The apparatus consisted in the arena (a plastic white box 50 × 35 × 45 cm) being placed on a lab bench with a webcam connected to the computer and was fixed on the wall, with objects of different colors and shapes (e.g., pyramid, cube, truncated sphere, cylinder, prism, and star) designed by SolidWorks software and 3D printed in polylactic acid by a Prusa-inspired 3D printer of our design. Three days before training (from day 21 to day 23), mice were habituated to the new context (empty arena and arena containing one or two objects) and allowed to freely explore it for 10 min. On day 24, mice, previously treated for 24 days with i.p. injections of antidepressants or vehicle, 45 min after the last injection of FLX or VTX, underwent the first trial (T1) of ORT consisting in exploring two identical objects (randomly chosen among our collection) placed in the central part of the box, equally distant from the perimeter. T1 lasted 10 min, a time sufficient to learn the task. The second trial (T2) was performed 24 h after T1 (day 25) to test memory retention for 10 min. Mice were presented with two objects, a “familiar” (i.e., the one used for T1) and a “novel” object. The latter was placed on the left or the right side of the box in a randomly but balanced manner, to minimize potential biases due to a preference for particular locations. To avoid olfactory cues, the objects and the apparatus were cleaned with 70% ethanol after each trial. Exploration, defined as the mouse pointing its nose toward the object from a distance not >2 cm (as marked by a reference circle), was manually evaluated by an investigator blind with respect to treatment. In particular, the following parameters were studied: i) discrimination index (D), calculated as “exploration of novel object minus exploration of familiar object/total exploration time,” and ii) total exploration time. We excluded from the analyses mice with a total exploration time < 5 s.

### Western Blot

Western blot analysis was performed as previously described (Caraci et al., 2015) on hippocampi of mice from the different experimental groups (*n* = 4 per group). Tissues were harvested at 4°C in radioimmunoprecipitation assay (RIPA) buffer, in the presence of a cocktail of protease inhibitors (Sigma-Aldrich, P2714), serine/threonine phosphatase inhibitors (Sigma-Aldrich, P0044), and tyrosine protein phosphatase inhibitors (Sigma-Aldrich, P5726), followed by sonication. Protein concentrations were determined by Bradford’s method using bovine serum albumin as a standard. After being blocked, membranes were incubated with the following primary antibodies, overnight at 4°C: rabbit anti-TGF-β1 (Abcam 92486, Cambridge, UK; 1:1,000), mouse anti-GAPDH (Millipore MAB374, Burlington, MA, USA; 1:1,000), rabbit anti-PSD-95 (3450S Cell Signaling Technology Inc., Danvers, MA, USA; 1:1,000), mouse anti-synaptophysin (SC-17750 Sunta Cruz Biotechnology Inc., CA, USA; 1:40.000), and rabbit anti-actin (A2066, Sigma-Aldrich, St Louis, MO; 1:5.000). Secondary goat anti-rabbit labeled with IRDye 680 (Li-COR Biosciences; 1:20.000) and goat anti-mouse labeled with IRDye 800 (Li-COR Biosciences; 1:20.000) were used at room temperature for 45 min. Hybridization signals were detected with the Odyssey Infrared Imaging System (LI-COR Biosciences). Western blot data were quantified by densitometry analysis of the hybridization signals in four different blots per experiment.

### Gene Expression Analysis by Real-Time RT-PCR

Gene expression analysis by quantitative qRT-PCR was performed as previously described (Caruso et al., 2019b) with slight modifications. In brief, the concentration of total RNA recovered by using RNeasy Mini Kit from 10 mg of hippocampus tissue was determined by measuring the absorbance at 260 nm with a Nano Drop^®^ ND-1000 (Thermo Fisher Scientific, Waltham, MA, USA). SuperScript III First-Strand Synthesis SuperMix (Thermo Fisher Scientific) was used to carry out the reverse transcription (100 ng of total RNA for each sample), by random priming. All samples were then quantified with a NanoDrop^®^ ND-1000, diluted to a final concentration of 25 ng/µL, and the gene expression was simultaneously measured for all the samples by using a 384-well plates and a LightCycler^®^ 480 System (Roche Molecular Systems, Inc., Pleasanton, CA, USA). The QuantiTect Primer Assays (Qiagen, Hilden, Germany) employed for gene expression analysis along with official name, official symbol, alternative titles/symbols, detected transcript, amplicon length, and primers catalogue number are shown in **Table 1**.

**Table 1:** List of primers used for quantitative real-time PCR (qRT-PCR).

Official name^#^	Official symbol	Alternative titles/symbols	Detected transcript	Amplicon length	Cat. no.^§^
Interleukin 1 beta	Il1b	Il-1b; IL-1beta; IL-1β	NM_008361 XM_006498795	150 682	QT01048355
Tumor necrosis factor	Tnf	DIF; Tnfa; TNF-a; TNFSF2; Tnlg1f; Tnfsf1a; TNFalpha; TNF-alpha; TNF-α	NM_013693 NM_001278601	112 bp 112 bp	QT00104006
Interleukin 4	IL4	Il-4; BSF-1	NM_021283	132 bp	QT02418311
Transforming growth factor, beta 1	Tgfb1	Tgfb; Tgfb-1; TGFbeta1; TGF-beta1	NM_011577	145 bp	QT00145250
Glyceraldehyde-3-phosphate dehydrogenase	Gapdh	Gapd	NM_008084 XM_001003314 XM_990238 NM_001289726	144 bp	QT01658692

^#^ https://www.ncbi.nlm.nih.gov/gene/

^§^ https://www.qiagen.com/it/shop/pcr/real-time-pcr-enzymes-and-kits/two-step-qrt-pcr/quantitect-primer-assays/

For each sample amplification, performed in quadruplicate, a total reaction volume of 10 μL, consisting of 6 μL of amplification mixture (5 μL PCR Master Mix + 1 μL specific primers) plus 4 μL of cDNA (100 ng), was used. Amplification conditions and fluorescence data collection included a first cycle at 95°C (15 min) followed by 50 cycles at 94°C (15 s), an annealing step at 56°C (30 s), and a final cycle at 72°C (30 s). As a negative control, a reaction in absence of cDNA (no template control, NTC) was performed. The relative RNA expression level for each sample was calculated using the 2^−ΔΔCT^ method by comparing the threshold cycle (CT) value of the gene of interest with the CT value of our selected internal control (GAPDH gene).

### Statistics

All experiments were blind with respect to treatment. Data were expressed as mean ± standard error mean (SEM). Statistical analysis was performed using dedicated software (GraphPad Prism, La Jolla, CA; Systat 9 Software, Chicago, IL). The within-group comparison was performed by a one-way analysis of variance (ANOVA). The *post hoc* Bonferroni test was used for multiple comparisons. One-sample *t*-test was used to compare D index with zero in ORT.

### Study Approval

The study was authorized by the Institutional Animal Care and Use Committee (IACUC) of the University of Catania and by the Italian Ministry of Health (DDL 26/2014 and previous legislation; OPBA Project #266/2016). Animal care followed Italian (D.M. 116192) and EEC (O.J. of E.C.L 358/1 12/18/1986) regulations on protection of animals used for experimental and scientific purposes.

## Results

### Fluoxetine and Vortioxetine Showed Similar Antidepressant Efficacy in Young Mice

**Figure 1:**
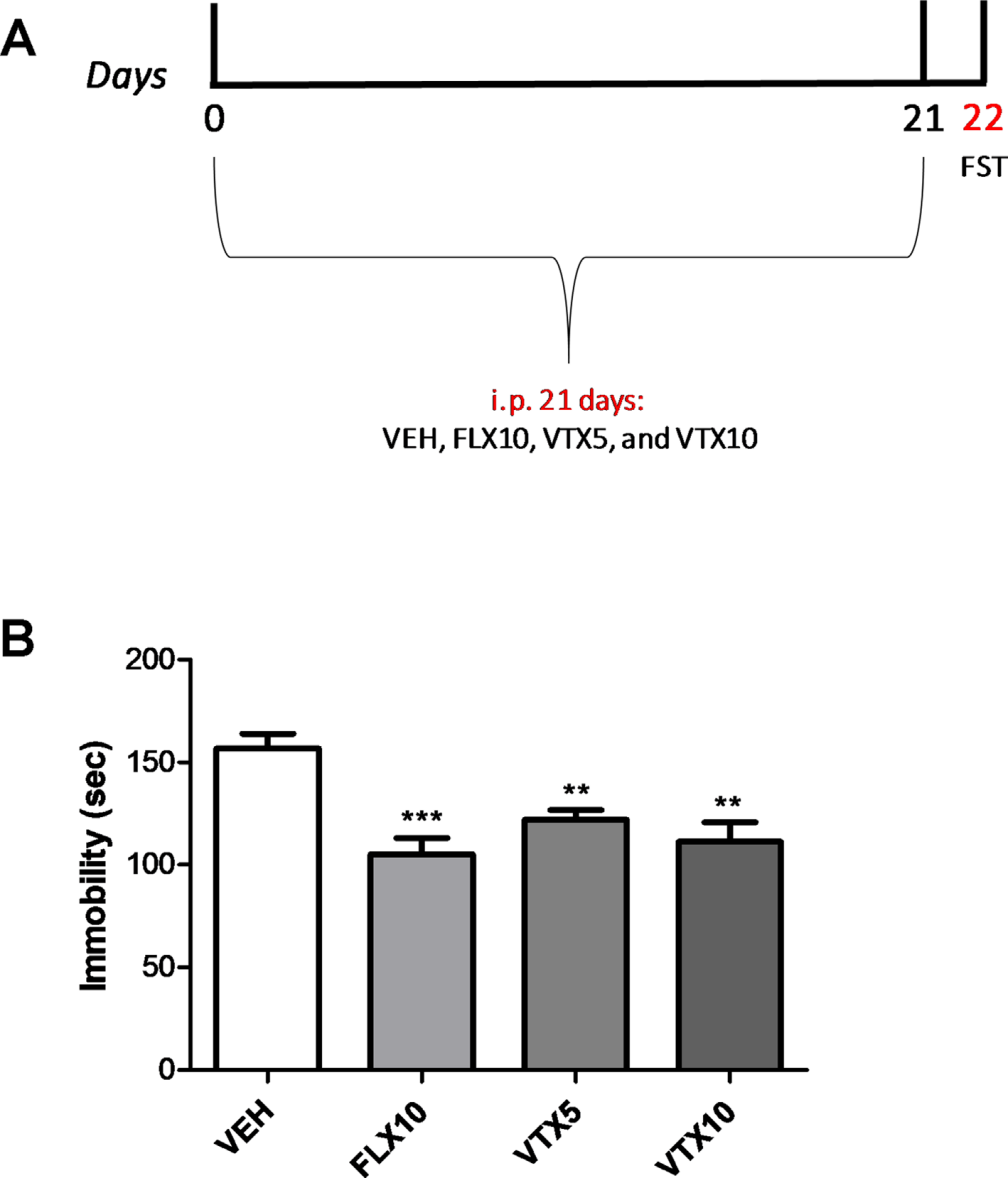
Vortioxetine decreases depressive-like behavior in a concentration-dependent manner. Forced swim test (FST), carried out to evaluate the depressive-like behavior, was performed the day after the last injection. VEH = vehicle (i.p.), FLX10 = fluoxetine 10 mg/kg, (i.p.), VTX5 = vortioxetine 5 mg/kg (i.p.), and VTX10 = vortioxetine 10 mg/kg (i.p.) were administered chronically for 21 days. i.p. = intraperitoneal injection. **(A)** Schematic representation of the experimental design. **(B)** Immobility time displayed by groups treated with FLX10 (*n* = 9), VTX5 (*n* = 10), and VTX10 (*n* = 7) was significantly reduced if compared with that of vehicle-treated group (*n* = 9) over a 4-min test period. Immobility time measures are expressed in seconds. Data are shown as mean ± SEM. **p < 0.01, ***p < 0.001 vs. VEH; ANOVA among all: *F*_(3,31)_ = 10.04.

We first examined the effects of FLX and VTX on depressive-like behavior in the first cohort of mice (Experiment 1) in the FST, a well-established behavioral test used to evaluate the preclinical efficacy of antidepressant drugs (Castagné et al., 2011; Li et al., 2017). Depressive-like behavior was assessed at day 22 by scoring immobility time (expressed in seconds) for each animal (**Figure 1A**). As depicted in **Figure 1B**, both FLX and VTX, at the dose of 10 mg/kg, gave comparable results, reducing the immobility time [*p* < 0.001 and *p* < 0.01 for FLX and VTX vs. vehicle (VEH), respectively]. VTX was able to significantly reduce the immobility time also at the dose of 5 mg/kg (*p* < 0.01 vs. VEH).

### Fluoxetine and Vortioxetine Prevented Memory Retention Loss and Depressive-Like Behavior Induced by Aβ Oligomers

**Figure 2:**
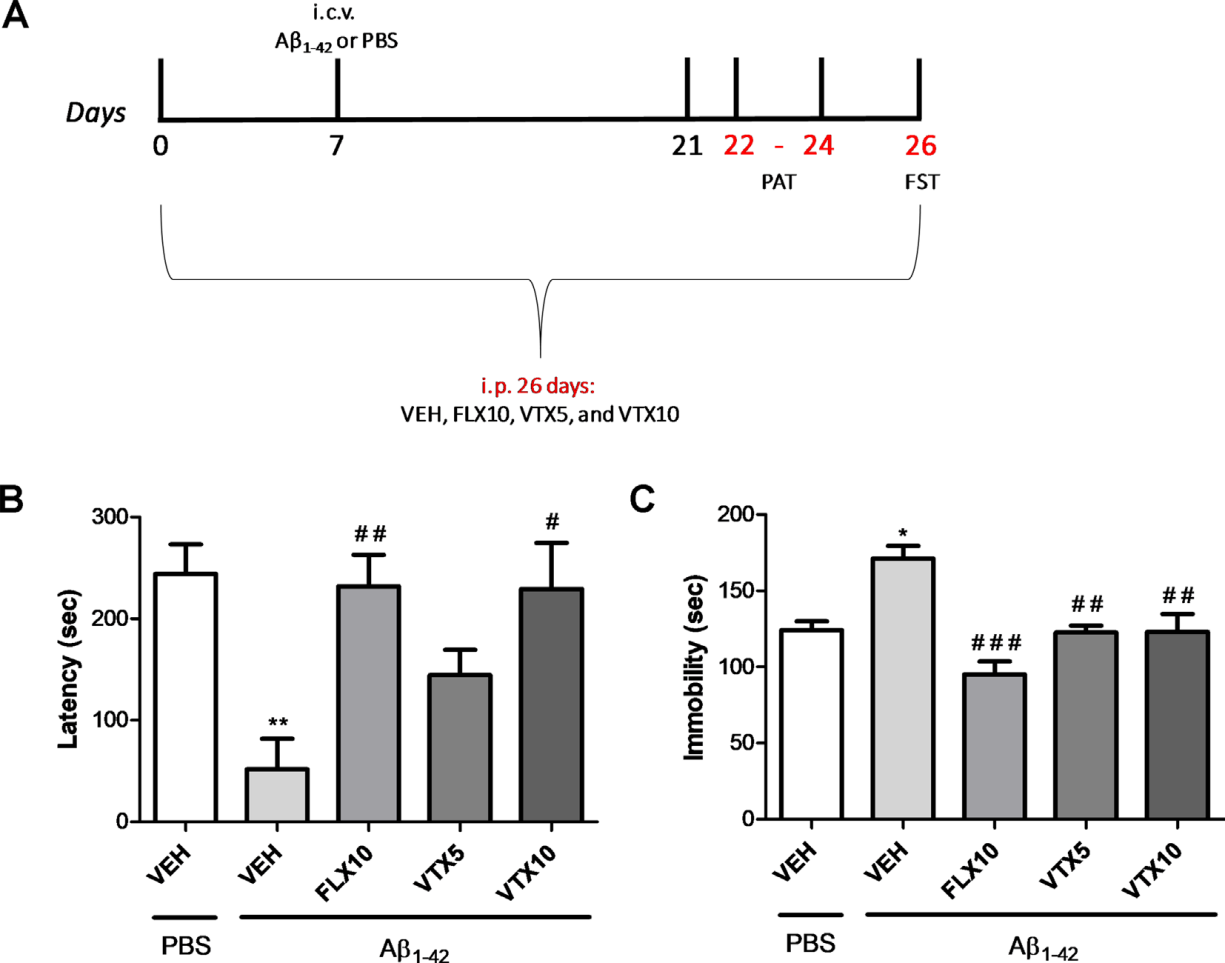
Vortioxetine decreases depressive-like behavior and memory impairment Aβ_1-42_-induced. Forced swim test (FST) and passive avoidance test (PAT) were used to evaluate depressive-like behavior and memory impairment, respectively. VEH, FLX10, VTX5, and VTX10 were administered chronically for 26 days. Sterile PBS or Aβ_1-42_ was administered i.c.v. 7 days after the first i.p. injection. i.c.v. = intracerebroventricular injection. **(A)** Schematic representation of the experimental design. **(B)** Latency time to re-enter the dark box during the retention test is expressed in seconds. **(C)** Immobility time measures are expressed in seconds. PBS + VEH (*n* = 8), Aβ_1-42_ + VEH (*n* = 5), Aβ_1-42_ + FLX10 (*n* = 7), Aβ_1-42_ + VTX5 (*n* = 7), and Aβ_1-42_ + VTX10 (*n* = 6). FST and PAT were performed on the same experimental animal groups. Data are shown as mean ± SEM. *p < 0.05, **p < 0.01 vs. PBS + VEH; ^#^ *p* < 0.05, ^##^ *p* < 0.01, ^###^ *p* < 0.001 vs. Aβ_1-42_ + VEH; *F*_(4,28)_ = 10.44 for **(B)** and *F*_(4,28)_ = 5.59 for **(C)**.

We then investigated the effects of FLX and VTX on the memory retention loss in mice treated with Aβ oligomers (second cohort of mice, Experiment 2). The treatment with antidepressants started 7 days before Aβ_1-42_ oligomers or PBS i.c.v. injection, and memory deficits were evaluated in the PAT with memory retention test after 24 days of chronic treatment with FLX or VTX (i.e., 17 days after Aβ injection, **Figure 2A**). As observed in our previous studies (Leggio et al., 2016), mice treated with Aβ_1-42_ showed a lower latency time in PAT than did vehicle-treated controls (*p* < 0.01 vs. VEH; **Figure 2B**). Interestingly, a chronic treatment with FLX (10 mg/kg) and VTX (10 mg/kg) was able to rescue Aβ-induced memory loss (*p* < 0.01 vs. Aβ + VEH and *p* < 0.05 vs. Aβ + VEH, respectively) (**Figure 2B**).

Depressive-like behavior was then evaluated in FST, in the same cohort of mice, 26 days after treatment with antidepressant drugs (19 days after Aβ injection; **Figure 2C**). We show, for the first time, that Aβ injection was able to induce a long-lasting significant increase in immobility time 19 days after i.c.v. Aβ injection (*p* < 0.05 vs. VEH). Chronic i.p. treatment with VTX or FLX, administered at the same dose of 10 mg/kg for 26 days, was able to revert Aβ_1-42_-induced depressive-like behavior (*p* < 0.001 and *p* < 0.01 for FLX and VTX vs. Aβ + VEH, respectively). Interestingly, VTX at the low dose of 5 mg/kg was also effective in preventing depressive-like behavior in Aβ-injected mice (*p* < 0.01 vs. Aβ + VEH).

### Fluoxetine and Vortioxetine Improved Object Recognition Memory in Aβ-Treated Mice

**Figure 3:**
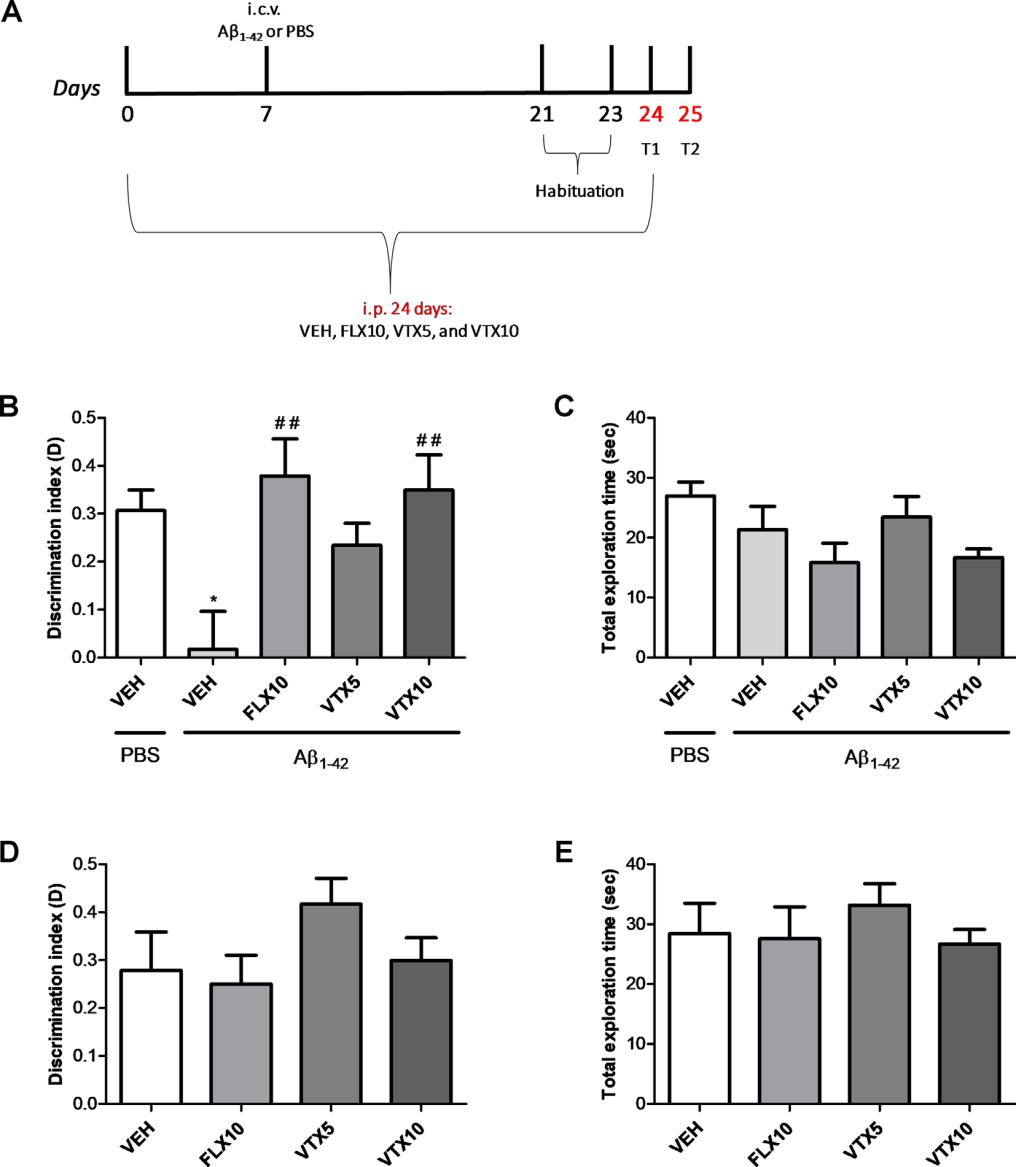
Vortioxetine reduces the Aβ_1-42_-induced impairment of recognition memory. Object recognition test (ORT) was used to evaluate recognition memory by assessing the discrimination index (D). **(A)** Schematic representation of the experimental design. **(B)** The impairment of recognition memory induced by i.c.v. administration of Aβ_1-42_ (*t*_(9)_ = 0.221, *p* > 0.05 for Aβ_1-42 _group vs. zero) is completely rescued by FLX10 and VTX10 treatments (ANOVA among all: *F*_(5,54)_ = 4.4; Bonferroni’s *p* < 0.05 between PBS + VEH and Aβ_1-42_ + VEH; *p* < 0.01 between Aβ_1-42_ + VEH and VTX10 and FLX10). **(C)** Total exploration time is similar among the different conditions (ANOVA among all: *F*_(5,54)_ = 2.274). PBS + VEH (*n* = 14), Aβ_1-42_ + VEH (*n* = 10), Aβ_1-42_ + FLX10 (*n* = 7), Aβ_1-42_ + VTX5 (*n* = 11), and Aβ_1-42_ + VTX10 (*n* = 11). **(D)** FLX10, VTX5, or VTX10 treatments *per se* do not modify discrimination index (*F*_(3,28)_ = 1.409) nor **(E)** total exploration time (*F*_(3,28)_ = 0.467). *p < 0.05 vs. PBS + VEH, ^##^ *p* < 0.01 vs. Aβ_1-42_ + VEH.

We then evaluated recognition memory by ORT, a task based on the natural tendency of rodents to explore unfamiliar objects, which depends upon integrity of the perirhinal cortex, the hippocampus, and the medial temporal lobe (Barker et al., 2007; Broadbent et al., 2009). We measured the exploration time of both the familiar and novel objects at T2, i.e., 24 h after training, in Aβ-injected mice; and we calculated the discrimination index (D = exploration of novel object minus exploration of familiar object/total exploration time) (**Figure 3A**). Aβ-injected mice, compared with vehicle-injected mice, showed an impairment of recognition memory, as they did not discriminate between the familiar and novel objects (*p* < 0.05; **Figure 3B**). Comparison of D with zero confirmed that Aβ-injected mice were not able to learn (*p* > 0.05). The chronic treatment with FLX (10 mg/kg) or VTX (10 mg/kg) was effective in rescuing Aβ-induced memory impairment (*p* < 0.01 vs. Aβ + VEH for both treatments). Results were not affected by differences in total exploration time between the animal groups (**Figure 3C**). Treatment with FLX or VTX *per se* did not modify discrimination index (**Figure 3D**) nor (**Figure 3E**) total exploration index.

### Molecular Mechanisms Underlying the Antidepressant and Procognitive Effects of Fluoxetine and Vortioxetine: A Key Role of TGF-β1

**Figure 4:**
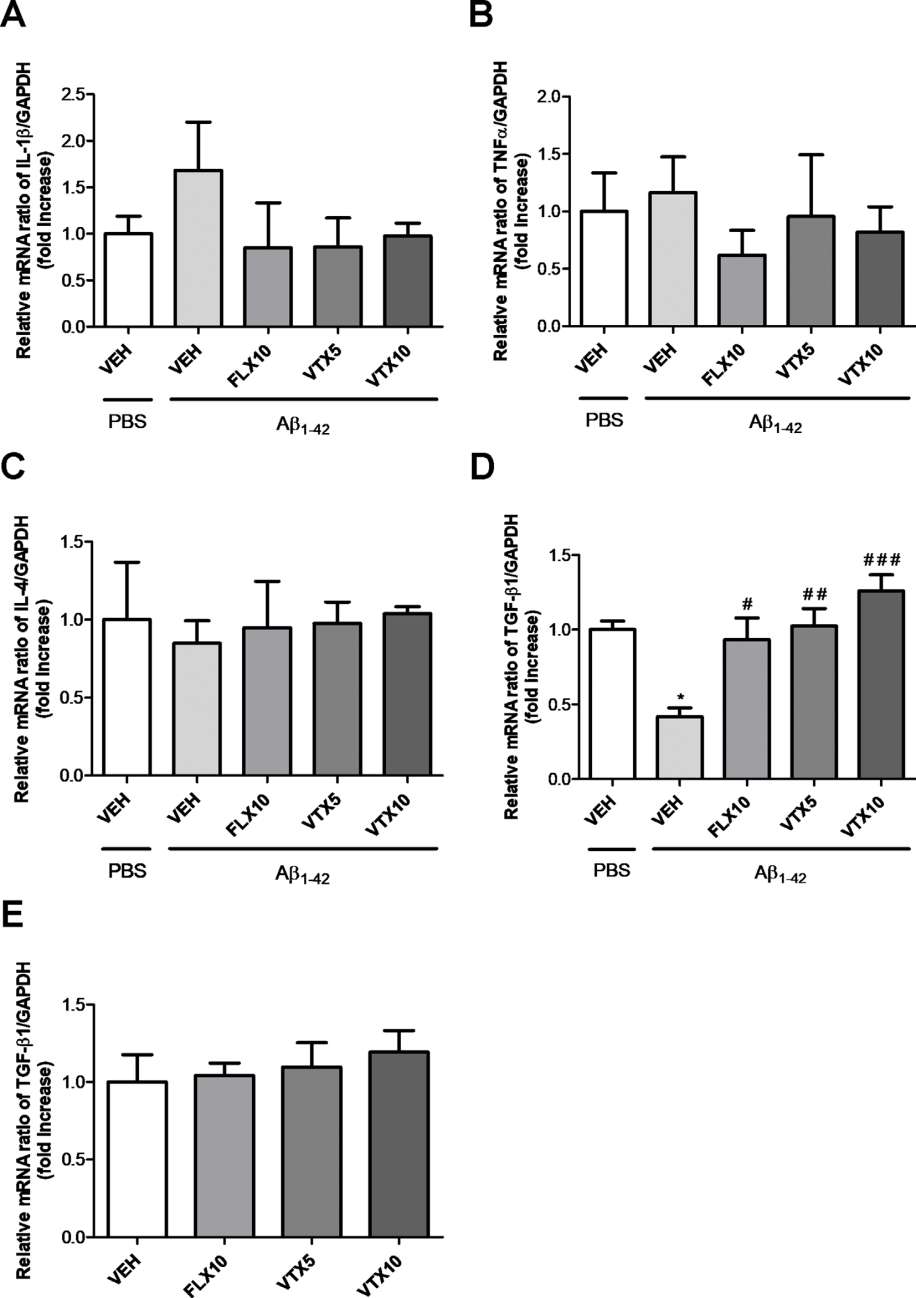
Fluoxetine and vortioxetine increase the expression of TGF-β1 mRNA. Effects induced by i.c.v. administration of Aβ_1-42 _(Aβ_1-42_ + VEH) in absence or presence of FLX10, VTX5, or VTX10 on IL-1β **(A)**, TNF-α **(B)**, IL-4 **(C)**, and **(D)** TGF-β1 mRNAs expression examined by qRT-PCR (Experiment 2). **(E)** Effects of drugs on TGF-β1 mRNA expression in absence of Aβ_1-42_ treatment (Experiment 3). The abundance of each mRNA of interest was expressed relative to the abundance of GAPDH-mRNA, as an internal control. As a negative control, a reaction in absence of cDNA (no template control, NTC) was performed. qRT-PCR amplifications were performed in quadruplicate. Data are shown as mean ± SEM. *p < 0.05 vs. PBS + VEH, ^#^ *p* < 0.05 vs. Aβ_1-42_ + VEH, ^##^ *p* < 0.01 vs. Aβ_1-42_ + VEH, ^###^ *p* < 0.001 vs. Aβ_1-42_ + VEH; *F*_(4,14)_ = 0.86 for **(A)**, *F*_(4,10)_ = 0.35 for **(B)**, *F*_(4,10)_ = 0.06 for **(C)**, *F*_(4,15)_ = 10.23 for **(D)**, and *F*_(3,19)_ = 0.35 for **(E)**.

Neuroinflammation plays a central role in the pathogenesis of depression (Bhattacharya et al., 2016) and AD (Businaro et al., 2018; Knezevic and Mizrahi, 2018). Previous studies have demonstrated that Aβ oligomers promote neuroinflammation and neurodegeneration in AD brain and in animal models of AD by eliciting the release of pro-inflammatory cytokines from microglia (Ledo et al., 2016; Businaro et al., 2018) and also by interfering with the synthesis of TGF-β1 (Diniz et al., 2017). We therefore examined the effects of Aβ_1-42_ oligomers i.c.v. injection on the mRNA levels of pro-inflammatory cytokines (IL-1β and TNF-α) and anti-inflammatory cytokines (IL-4 and TGF-β1) in the hippocampus (**Figure 4**), a brain area of primary relevance in the pathogenesis of depression (Villa et al., 2016). Aβ injection did not affect the expression level of IL-1β and TNF-α mRNA (**Figure 4A and B**), and the expression level of IL-4 (**Figure 4C**), whereas it induced a statistically significant decrease in the expression level of TGF-β1 mRNA in the hippocampus of Aβ-injected mice compared with vehicle-treated controls (*p* < 0.05 vs. VEH; **Figure 4D**). Interestingly, VTX at the low dose (5 mg/kg) was able to completely rescue hippocampal TGF-β1 mRNA levels compared with those in Aβ-injected mice (*p* < 0.01 vs. Aβ + VEH), and it further increased TGF-β1 mRNA levels at the dose of 10 mg/kg (*p* < 0.001 vs. Aβ + VEH). FLX at the dose of 10 mg/kg rescued hippocampal TGF-β1 mRNA levels with an efficacy comparable with that of VTX 5 mg/kg (*p* < 0.05 vs. Aβ + VEH). These antidepressant drugs *per se* did not increase hippocampal TGF-β1 mRNA (**Figure 4E**).

**Figure 5:**
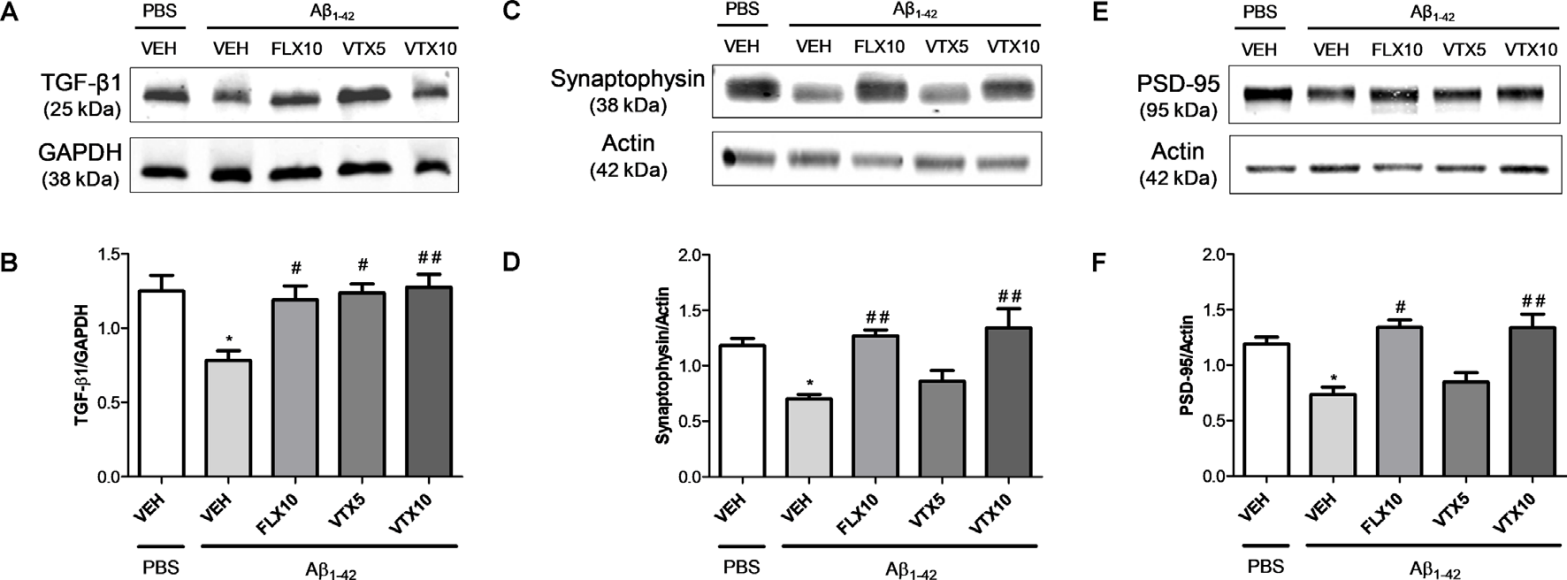
Fluoxetine and vortioxetine rescue TGF-β1, synaptophysin, and PSD-95 levels in Aβ_1-42_-treated mice. Effects induced by i.c.v. administration of Aβ_1-42_ (Aβ_1-42_ + VEH) in absence or presence of FLX10, VTX5, or VTX10 on TGF-β1, synaptophysin and PSD-95 levels examined by western blot. (A) Representative immunoblots of active TGF-β1 (about 25 kDa) in total protein extracts from hippocampus tissues. **(B)** Histograms refer to the means ± SEM of the densitometric values of active TGF-β1 bands normalized against GAPDH. Each experiment was repeated four times. *p < 0.05 vs. PBS + VEH, ^#^ *p* < 0.05 vs. Aβ_1-42_ + VEH, ^##^ *p* < 0.01 vs. Aβ_1-42_ + VEH; *F*_(4,15)_ = 5.91 for **(B)**. **(C)** Representative immunoblots of **synaptophysin** (about 38 kDa) in total protein extracts from hippocampus tissues. **(D)** Histograms refer to the means ± SEM of the densitometric values of **synaptophysin** bands normalized against actin. Each experiment was repeated four times. *p < 0.05 vs. PBS + VEH, ^##^ *p* < 0.01 vs. Aβ_1-42_ + VEH; *F*_(4,17)_ = 7.91 for **(D)**. **(E)** Representative immunoblots of **PSD-95** (about 95 kDa) in total protein extracts from hippocampus tissues. **(D)** Histograms refer to the means ± SEM of the densitometric values of **PSD-95** bands normalized against actin. Each experiment was repeated four times. *p < 0.05 vs. PBS + VEH, ^#^ *p* < 0.05 vs. Aβ_1-42_ + VEH, ^##^ *p* < 0.01 vs. Aβ_1-42_ + VEH; *F*_(4,17)_ = 8.21 for **(F)**.

TGF-β1 is an anti-inflammatory cytokine whose final activity is regulated not only at a transcriptional level but also at a post-transcriptional level and primarily regulated through the conversion of latent TGF-β1 to active TGF-β1 by a variety of proteases (Annes et al., 2003). Interestingly, western blot analysis carried out in the hippocampus of these mice confirmed that i.c.v. Aβ injection was able to induce a significant decrease of active TGF-β1 levels (*p* < 0.05 vs. PBS + VEH) and, most importantly, that both FLX and VTX (at both doses) were able to completely rescue hippocampal TGF-β1 levels when compared with those in Aβ-injected mice treated with vehicle (*p* < 0.01 vs. Aβ + VEH for FLX and VTX at 5 mg/kg; *p* < 0.001 vs. Aβ + VEH for VTX at 10 mg/kg; **Figure 5A and B**). Since it is known that TGF-β1 protects synapses against Aβ oligomers toxicity (Diniz et al., 2017), we examined the expression levels of two established synaptic protein markers, synaptophysin and PSD-95, in the hippocampus of Aβ-injected mice. Aβ injection significantly decreased both synaptophysin (**Figure 5C and D**) and PSD-95 levels (**Figure 5E and F**) (*p* < 0.05 vs. PBS + VEH); and, interestingly, both FLX and VTX (at 10 mg/kg) rescued hippocampal synaptophysin (*p* < 0.01 vs. Aβ + VEH for FLX and VTX at 10 mg/kg) and PSD-95 (*p* < 0.05 vs. Aβ + VEH for FLX and *p* < 0.01 vs. Aβ + VEH for VTX at 10 mg/kg) levels when compared with those in Aβ-injected mice treated with vehicle.

## Discussion

In this paper, we have demonstrated for the first time that a long-term treatment with fluoxetine (10 mg/kg/day) or with the multimodal antidepressant vortioxetine (5 and 10 mg/kg/day) was able to prevent the loss of memory and the Aβ_1-42_ oligomer-induced depressive-like phenotype with a key contribute played by TGF-β1 in the mouse hippocampus.

We have used a non-Tg model of AD obtained by i.c.v. injection of Aβ_1-42 _oligomers, known to play a primary role in synaptic loss and progressive cognitive decline in AD (Ferretti et al., 2012; Klein, 2013). Synthetic human Aβ_1-42 _oligomers were prepared according to the original protocol of Klein’s group as modified and characterized in Giuffrida et al. (2009). An open question in the field remains to establish whether Aβ_1-42 _oligomers can induce transient or long-term memory deficits in mice (Balducci and Forloni, 2014; Epelbaum et al., 2015). Different groups have demonstrated that, in the field of translational neuropharmacology, this model represents a simple and reliable paradigm, useful to investigate the molecular mechanisms through which Aβ oligomers interfere with cognitive processes and finally to test the efficacy of new therapeutic approaches (Balducci and Forloni, 2014). We have adopted this non-Tg AD model because we know from our previous work that i) the amount of injected oligomers reaches a cerebral concentration comparable with the concentration of soluble Aβ observed in AD brains, e.g., close to 1 µg/g (Leggio et al., 2016); and ii) i.c.v. injection of Aβ induces a memory deficit that persists for 14–21 days, as assessed by using two well-validated tasks in AD field, the passive avoidance task and the object recognition test (Leggio et al., 2016). We used this non-Tg model of AD to study the neurobiological links between depression and AD and the role of Aβ oligomers in the pathophysiology of amyloid-related depression, a recently identified clinical phenotype characterized by a low response to “monoaminergic antidepressants in depressed patients with an high risk to develop AD” (Li et al., 2017). Mimicking this clinical phenotype in rodents is a difficult challenge (Nyarko et al., 2019) but also an essential step to improve drug discovery processes in AD and explore the disease-modifying potential of antidepressant drugs in AD (Caraci et al., 2018).

Previous studies have been conducted in rodents where a depressive-like phenotype was detected by FST only 7 days after a single Aβ injection in rats (Colaianna et al., 2010; Schiavone et al., 2017) or 24 h after Aβ infusion in mice (Ledo et al., 2016). In the present work, we demonstrate for the first time that Aβ injection can induce a long-lasting depressive-like phenotype, with a significant reduction in immobility time detectable with FST until 19 days after Aβ injection (**Figure 2C**). Interestingly, this depressive-like phenotype co-exists in our Aβ-injected mice with a severe impairment of reference memory (assessed by PAT) (**Figure 2B**) and object recognition memory (assessed by ORT) (**Figure 3A and B**). In the present work, only one memory test was conducted in each cohort of mice (second and third) to minimize potential effect of behavioral testing on FST. Future studies should be conducted in the same model to assess whether depressive-like phenotype precedes the onset of cognitive deficits as recently observed in late-life depressed patients with an increased risk to develop AD (Chung et al., 2015; Yasuno et al., 2016).

In the present work, we measured the antidepressant-like efficacy of fluoxetine and vortioxetine in FST, in the second cohort of Aβ-injected mice, after a 26-day treatment. Drug doses for both fluoxetine and vortioxetine were chosen to reach a reliable occupancy of SERT in brain, as reported in previous studies (Pehrson et al., 2015). For the present study, we selected these specific antidepressants because fluoxetine is a SSRI known to revert cognitive deficits in different transgenic animal models of AD (Wang et al., 2014; Jin et al., 2016; Ma et al., 2017; Sun et al., 2017), and it is also able to rescue memory deficits in MCI patients (Mowla et al., 2007), while vortioxetine is a novel multimodal antidepressant endowed with strong pro-cognitive effects in preclinical models of depression (Pehrson et al., 2015) with a high clinical efficacy in the treatment of elderly patients with late-life depression and cognitive symptoms, a clinical subgroup that shows an increased risk to develop AD (Lauriola et al., 2018).

Interestingly, when comparing the effects of a chronic treatment (26 days) of fluoxetine and vortioxetine in our non-Tg AD model, we found for the first time that these two drugs have a similar preclinical efficacy at a dose of 10 mg/kg/day in preventing memory deficits, as assessed by PAT and ORT. Other studies have shown that fluoxetine can impair recognition memory in rats (Valluzzi and Chan, 2007) and in middle-aged mice (Castañé et al., 2015; Li et al., 2017), whereas vortioxetine does not affect object recognition memory in middle-aged mice (Li et al., 2017) but significantly improves the performance in this task in different animal models of cognitive dysfunction (Westrich et al., 2015; Pehrson et al., 2018). Surprisingly, 5 mg/kg vortioxetine exerted a significant antidepressant effect as detected in FST (without a further increase at a dose of 10 mg/kg), which was comparable with that of fluoxetine 10 mg/kg. Considering that vortioxetine at the dose of 5 mg/kg nearly saturates all 5-HT_3_ receptors, but only partially occupies the SERT (Sanchez et al., 2015), these data seem to suggest an increased, and probably SERT-independent, antidepressant efficacy of vortioxetine compared with fluoxetine in our model of amyloid-related depression. We cannot exclude that the young age of our cohorts of mice can affect our results in behavioral tests, but we should also consider that in this study we have adopted a secondary prevention strategy to prevent the onset of amyloid-related depression, starting the treatment with antidepressants 7 days before Aβ injection. This approach was also settled moving from the evidence that second-generation antidepressants, such as fluoxetine, exert relevant neuroprotective effects *in vitro* in experimental models of Aβ-induced neurodegeneration (Caraci et al., 2016; Caraci et al., 2018). We also believe that this approach might be helpful in the future to assess the disease-modifying efficacy of antidepressants in animal models of AD, independently from their symptomatic efficacy against the depressive-like phenotype.

To understand the molecular mechanisms underlying the precognitive and antidepressant effects of vortioxetine and fluoxetine, we focused on neuroinflammatory phenomena in the hippocampus of Aβ-injected mice, because previous studies in the same model found aberrant TNF-α signaling with increases in hippocampal levels of TNF-α 24 h after Aβ infusion (Ledo et al., 2016). In order to correlate the preclinical efficacy of antidepressants with the effects on neuroinflammatory phenomena, we examined the mRNA levels of different pro-inflammatory (IL-1β and TNF-α) and anti-inflammatory (IL-4 and TGF-β1) cytokines in the hippocampus of the second cohort mice only after completing behavioral tests (26 days). We did not detect a significant increase in hippocampal levels of TNF-α and IL-1β (**Figure 4A and B**), but we found a significant decrease in hippocampal levels of TGF-β1 (**Figure 4D**), further confirmed by western blot analysis (**Figure 5A and B**). Our data are in accordance with a previous study conducted in 3-month-old male Swiss mice, where reduced TGF-β1 levels were found in the hippocampus 24 h after Aβ injection (Diniz et al., 2017). Interestingly, we found that the deficit of hippocampal TGF-β1 is a long-lasting molecular marker associated with depressive-like phenotype and memory deficits in our non-Tg model of AD. TGF-β1 is an anti-inflammatory cytokine that exerts neuroprotective effects in different models of amyloid-induced neurodegeneration (Caraci et al., 2008; Caruso et al., 2019a; reviewed by Caraci et al., 2011). We have recently identified a key role for TGF-β1 in recognition memory formation, demonstrating that it is essential for the transition from early to late long-term potentiation (Caraci et al., 2015). Deficit of TGF-β1 signaling is a primary event in AD pathogenesis, and a reduced expression of type 2 TGF-β1 receptor specifically correlates with cognitive decline in early AD patients (Tesseur et al., 2006). TGF-β1 plays a key role in synaptic plasticity (Caraci et al., 2015), and it also protects synapses against Aβ oligomers toxicity (Diniz et al., 2017). Interestingly, we found, in our non-Tg model of AD, a significant reduction of the synaptic proteins synaptophysin and PSD-95 paralleling the deficit of TGF-β1 detected in the hippocampus of Aβ-injected mice. Aβ oligomers are known to exert synaptotoxic effects (Musardo and Marcello, 2017), and our data are in accordance with previous studies where i.c.v. Aβ injection in mice caused both memory deficits and a significant decrease of PSD-95 and synaptophysin levels in the hippocampus (Morroni et al., 2016; Wu et al., 2018). In the present work, for the first time, we found a correlation between the synaptotoxic effects of Aβ oligomers and the deficit of TGF-β1 in the hippocampus of Aβ-injected mice.

The deficit of TGF-β1 signaling has been hypothesized to contribute to inflammaging and cognitive decline in both depression and AD (Caraci et al., 2018). The +10 CC genotype of TGF-β1 gene, which affects the levels of expression of TGF-β1, is associated with depressive symptoms in AD (>5-fold risk) (Caraci et al., 2012), and an impairment of TGF-β1 signaling can promote the onset of a depressive-like phenotype in mice (Depino et al., 2011). TGF-β1 plasma levels are reduced in MDD patients, correlate with depression severity, and significantly contribute to treatment resistance in MDD patients (Musil et al., 2011; Caraci et al., 2018), a clinical subgroup with an increased risk to develop AD (Chung et al., 2015; Li et al., 2017).

Our work identified for the first time a selective deficit of TGF-β1 in a non-Tg model of AD that mimics what was observed in AD brain and, most importantly, showed that vortioxetine (5 mg/kg) and fluoxetine (10 mg/kg) completely rescue hippocampal TGF-β1 levels. Interestingly, fluoxetine and vortioxetine completely rescued hippocampal synaptophysin and PSD-95 levels in Aβ-injected mice only at the dose of 10 mg/kg, suggesting a protective effect of these drugs against the synaptotoxic effects of Aβ oligomers. Fluoxetine was known to induce TGF-β1 release from cortical astrocytes (Caraci et al., 2016), but this is the first demonstration that a chronic treatment with the multimodal antidepressant vortioxetine promotes TGF-β1 synthesis at hippocampal level in an animal model of amyloid-related depression. Future studies should be conducted in transgenic animal models of AD to assess whether fluoxetine or vortioxetine can prevent amyloid-induced depression and cognitive deficits by rescue of TGF-β1 signaling.

Overall, our data, obtained in a non-Tg model of AD, indicate that a deficit in TGF-β1 might represent one of the neurobiological links between depression and AD and also that rescue of TGF-β1 signaling with second-generation antidepressants might represent a new pharmacological strategy to prevent both amyloid-induced depression and cognitive decline in AD.

## Data Availability Statement

All datasets generated for this study are included in the manuscript and the supplementary files.

## Ethics Statement

The study was authorized by the Institutional Animal Care and Use Committee (IACUC) of the University of Catania and by the Italian Ministry of Health (DDL 26/2014 and previous legislation; OPBA Project #266/2016). Animal care followed Italian (D.M. 116192) and EEC (O.J. of E.C.L 358/1 12/18/1986) regulations on protection of animals used for experimental and scientific purposes.

## Author Contributions

FC gave substantial contributions to the conception and design of the work. ST, FG, MT, MG, AF, NM, GS, and GC performed the experiments. FC, SS, DP, and GL analyzed the data. GL, FT, AP, SS, DP, and FD participated in the design of the study. FC and GL drafted the work. All authors approved the version to be published.

## Funding

This research was conducted with the unrestricted support of Lundbeck.

## Conflict of Interest Statement

The authors declare that the research was conducted with the unrestricted support of Lundbeck according to the MTA N.417394 signed by University of Catania (Department of Drug Sciences) and H. Lundbeck A/S and Lundbeck Italia S.p.a.

In this study the funders had no role in study design, data collection and analysis, decision to publish, or preparation of the manuscript.

The authors declare that the research was conducted in the absence of any commercial or financial relationships that could be construed as a potential conflict of interest.

The handling editor declared a past co-authorship with one of the authors, SS.
